# An Impact Study of the Design of Exergaming Parameters on Body Intensity from Objective and Gameplay-Based Player Experience Perspectives, Based on Balance Training Exergame

**DOI:** 10.1371/journal.pone.0069471

**Published:** 2013-07-26

**Authors:** Tien-Lung Sun, Chia-Hsuan Lee

**Affiliations:** Department of Industrial Engineering and Management, Yuan-Ze University, Taoyuan, Taiwan, ROC; ICREA-University of Barcelona, Spain

## Abstract

Kinect-based exergames allow players to undertake physical exercise in an interactive manner with visual stimulation. Previous studies focused on investigating physical fitness based on calories or heart rate to ascertain the effectiveness of exergames. However, designing an exergame for specific training purposes, with intensity levels suited to the needs and skills of the players, requires the investigation of motion performance to study player experience.

This study investigates how parameters of a Kinect-based exergame, combined with balance training exercises, influence the balance control ability and intensity level the player can tolerate, by analyzing both objective and gameplay-based player experience, and taking enjoyment and difficulty levels into account.

The exergame tested required participants to maintain their balance standing on one leg within a posture frame (PF) while a force plate evaluated the player's balance control ability in both static and dynamic gaming modes. The number of collisions with the PF depended on the frame's travel time for static PFs, and the leg-raising rate and angle for dynamic PFs. In terms of center of pressure (COP) metrics, significant impacts were caused by the frame's travel time on MDIST-AP for static PFs, and the leg-raising rate on MDIST-ML and TOTEX for dynamic PFs. The best static PF balance control performance was observed with a larger frame offset by a travel time of 2 seconds, and the worst performance with a smaller frame and a travel time of 1 second. The best dynamic PF performance was with a leg-raising rate of 1 second at a 45-degree angle, while the worst performance was with a rate of 2 seconds at a 90-degree angle.

The results demonstrated that different evaluation methods for player experience could result in different findings, making it harder to study the design of those exergames with training purposes based on player experience.

## Introduction

Exergaming is envisioned to be capable of merging fun from the gaming side with potential health and wellness benefits, enabling players to undertake physical exercise in a pleasant and interesting environment [1]. Exergaming allows the player to enjoy the game in an interactive manner by means of visual stimulation, and thus makes them perform physical activities. In addition, exergaming is not limited to time, space, or any issues regarding gaming partners. Individuals are free to undertake physical exercise at their own pace by immersing themselves in the exergame, in terms of both their visual sense and body. Confronted with a game scene that requires full-body movement, players are able to interact with the game, which in turn responds to the player's movement [1]–[4]. In terms of the benefits that it has on health, exergaming integrates physical exercise with video games, therefore significantly inspiring enthusiasm for undertaking exercise and improving the fun of exercise [2]–[3]. Not only do exergames enable players to do physical exercise at home with great enjoyment, they also overcome any time and space limitations that are associated with traditional exercise.

Equipment for exergames can be classified into four main categories; exercise bikes, foot operated pads, motion sensors, and other physically interactive games [4]–[5]. Among them, motion sensors have been widely available on the market, such as Sony PlayStation Move [6], Nintendo Wii [7], and Kinect Xbox [8]. Kinect does not require any hand-held remote control device, but instead it uses 3D depth sensing technology to detect the movement and position of the player. This is different from the aforementioned Wii and Move, both of which require an infrared remote control device to communicate with the master machine for positioning and movement detection. Although the infrared remote controller has an integrated three-axis accelerometer, its sensing effectiveness is limited due to the fact that the player can play with the remote controller in a “lazy” manner, and “cheat” the sensing device by undertaking the minimum amount of physical exercise [9]. To play the Wii balance board exergame, for example, the only thing that the player needs to do is to lift their feet slightly above the balance board in order to control the avatar.

Previous studies on designing effective balance-training programs based on virtual reality technology as well as exergaming can be divided into two streams. The first stream is focused on investigating the impact of different display patterns on the balance control performance of the experimental subject, whereas the second stream is mainly focused on examining how the design of game parameters influences the performance of balance control. To elaborate on this in greater detail, the first research perspective is normally based on displaying the virtual reality scene to the subject to evaluate the player experience for different display settings, where no interactions with objects in the scene take place [10]–[12]. The second perspective studies the balance control performance for different game parameters by varying the parameter settings and assessing the player's body-sway level [13]–[14].The body-sway level is represented by the maximum sway distance of the body along the anterior-posterior and the mediolateral directions, if the experimental subject is balanced. However, the impact of changes in game parameters followed by intensity changes in visual stimulation on the balance control ability of the experimental subject has not been studied. The attractiveness of a game is that it is fun to play. A game needs to provide multiple difficulty levels for players, and simultaneously ensure that the level of challenge increases as the player progresses through game to increase their skill level [15]. Exergaming takes the individuality of the player into account and enables the player to customize the game to meet their own individual preference, which is an indispensable factor to add more fun in the form of entertainment as well as more motivation for playing. Many previous studies have sorted the players into classes based on theories of psychology [16]–[18], and proposed the direction in which the game would be developed. However, the objective of this study is to investigate the impact of game parameters on player experience based on an example of a balance exergame, which has been addressed in few existing works.

Sinclair et al. [4] have proposed a model, referred to as the dual flow model, for the development of exergames. They claim that both the attractiveness and the effectiveness, which are seen as two critical factors in the success of exergames, need to be taken into account in order to enable the player to reach the state of total engagement in an activity, known as the “flow” state. The attractiveness of an exergame is a psychological model that balances the player's skill with perceived challenge, where the difficulty level of the activity is continually adjusted and matched with the level of skill in order to maintain the interest of the player. However, exergaming is different from playing an ordinary video game with a mouse and keyboard, in that it is played in an interactive manner. This characteristic results in another key construct of the dual flow model, referred to as effectiveness, which is the physiological counterpart with respect to the aforementioned psychological model. Effectiveness deals with the physical balance between the body's skill in tolerating exercise and the challenge of the exercise on the player's body, referred to as fitness and intensity, respectively. When the objective of playing an exergame is shifted from simply energy consumption to some specific training purposes, such as balance and upper extremity training games, representation of the intensity level is also shifted from energy consumption indicators, such as heart rate and blood oxygen level, to some indicators that are more relevant to the training purpose. For balance training, for example, intensity is represented by the displacement and the rate of the center of gravity shift, while fitness is evaluated based on the ability to shift the center of gravity. For upper extremity training, the exercise intensity is obtained from the displacement and the rate of holding up the upper limbs, and the fitness level is represented by the ability to raise the upper extremities. In other words, in order to design a customized exergame for balance training purposes, the ability to shift the center of gravity needs to be considered in the player model, and similarly, the shift displacement and rate need to be measured to evaluate player experience.

Current assessment methods of player experience for exergaming can be classified into subjective, objective, and gameplay-based modeling approaches, respectively [19]. The subjective approach considers only first person reports (also known as self-reports) and excludes those reports expressed indirectly by experts or other external observers [2]–[3], [20]–[22]. Objective player experience modeling, unlike the aforementioned method, is based on monitoring bodily alterations, which may greatly assist in recognizing and synthesizing the responses of the player, for example, including the performance of movements while playing the game [10]–[14], [21]–[25]. Gameplay-based player experience modeling, in comparison to the first two approaches, takes into account player actions and real-time preferences, among which the player's performance in the game is one of the main considerations [2], [3], [13], [20], [21], [23], [25]. This study, using a Kinect based exergame as an example, aims to improve the effectiveness of exergaming, with the ultimate goal of improving the deployment of exergames for home health and fitness purposes. Feedback from self-reports cannot be used as a standardized approach for player experience modeling due to its inherent limitations in terms of high subjectivity, which is readily affected by past experience, cognition, and self-perception of the player. As exergaming is highly based on human-computer interactions, where the player needs to make movements visually stimulated by the virtual scene, previous studies have already used the motion capture system with a high-speed digital camera and a force plate to collect the player's movement data. These studies, however, have not addressed in detail how different game parameters impose an impact on player experience. Because the optimum difficulty level as well as other game settings vary from individual to individual, this study, based on a balance training exergame, attempts to investigate the impact of different game parameters on the balance control ability and the fitness level of the exercise participant via both objective and gameplay-based player experience modeling approaches.

To sum up, this study deploys an exergame for balance training and studies the impact of variations in game parameters on the balance control skill of the player. In addition, the work also investigates the design of difficulty levels for merging physical exercise with a video game. By designing multiple difficulty levels, players are able to choose a suitable level in terms of the balance between intensity and fitness, the results of which will be used as feedback to improve the design of balance exergames.

## Materials and Methods

### Kinect exergame of balance training

**Figure 1:**
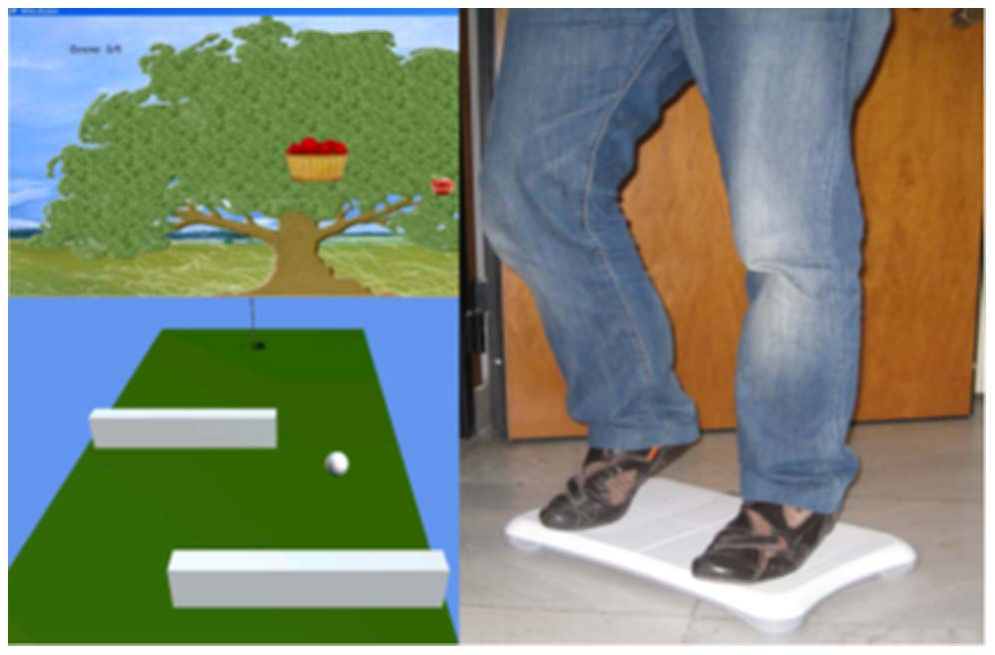
Balance game with Wii Balance Board (Billis et al., 2010).

Previous studies on balance games combined virtual game scenes with a Nintendo Wii Balance Board to enable intuitive interactions between the player and the game itself [26]–[29]. A Wii Balance Board offers the ability to communicate with its four sensors' raw data and Center of Pressure (COP) value through Bluetooth wireless communication protocol [26]. The COP represents a weighted average of all the pressures over the surface of the area in contact with the ground [13]. For example, in the work presented by Billis et al., as shown in Figure 1, the balance game comprises two game therapies. The first game is a well-known golf game. The user has to move his or her body in order to direct the ball past the barriers and get it in the hole. The second game requires seniors to move their body to control the movement of a basket. The aim of this game is to move the basket to catch as much fruit as possible. The fruits appear in random positions on the game screen at various time intervals. The game difficulty level is therefore based on parameters such as the size of the barrier, travel time, and speed. By integrating Kinect into the design, this work is an extension of these existing game design theories.

Widely used methods for balance training include the one-leg stand test, the get-up and go test, and the functional reach test [13]–[14], [19]–[20], [30]. Because standing on one leg is the most common posture in daily life, such as when navigating the stairs, stepping over obstacles, and in normal walking, and people are most likely to fall down while standing on one leg as a result of a shift in the center of gravity, many previous studies have used the one-leg stance as the posture for balance training [31]–[33]. Therefore, the objective of this work is to examine the impact of difficulty levels and game parameters on balance control performance of the experimental subject depending on the intensity of exercises when assuming the one-leg stance posture, and to study the design of the intensity level of exergames.

**Figure 2:**
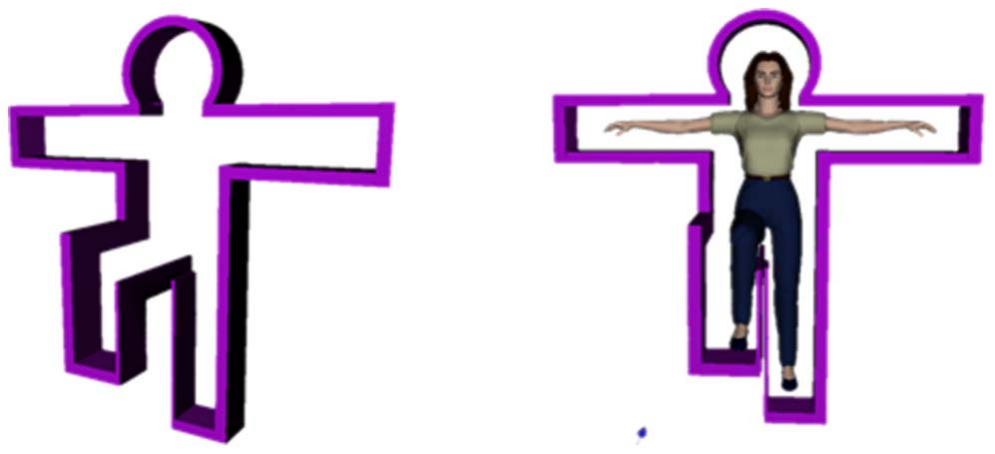
Diagram of the posture frame. (a)Posture frame with larger width. (b) Posture frame with smaller width.

**Figure 3:**
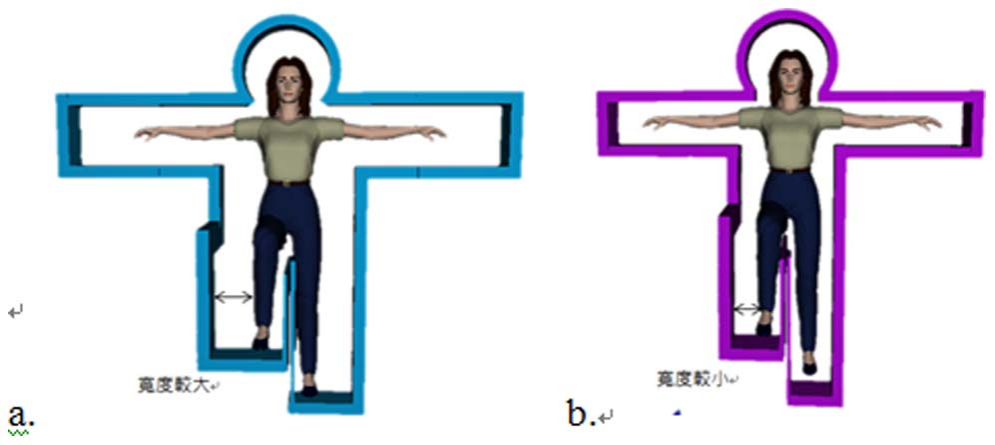
Diagram of the frame width.

**Figure 4:**
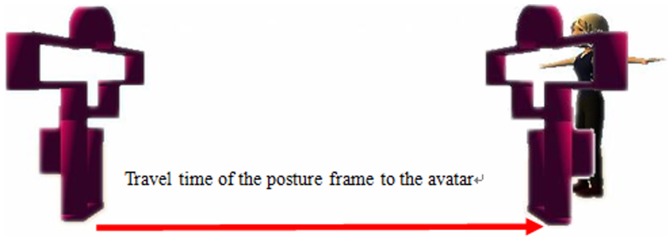
Diagram of the design of posture frame travel time to the avatar. (a) A raising angle of 45 degrees. (b) A raising angle of 90 degrees.

**Figure 5:**
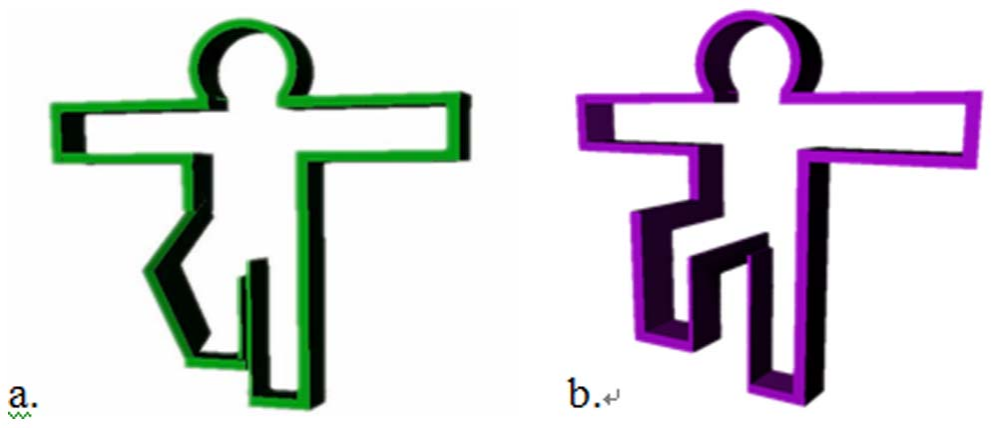
Diagram of the design of leg-raising angle.

As previously mentioned, this work uses a Kinect-based exergame for balance training, which is based on a posture frame that makes the player stand on one leg, as illustrated in Figure 2. The posture frame will appear on the screen and gradually approach the avatar in the virtual scene. The player then needs to make the one-leg stance posture to make the avatar pass through the posture frame without colliding with it. Although the posture frame is able to serve as a visual stimulation and forces the player to stand on one leg, different designs of the posture frame might impose an impact on the balance control ability of the participant. This study uses a static posture frame (PF) as studied by Hsu [34], Lai et al. [35], and a dynamic PF taken from the work by Chang [36], and evaluates the player experience in the case of different designs of visual stimulation and challenge levels, in order to learn the impact of game parameters on the intensity of exercise. For the static PF, the studied parameters are the offset of the PF and the travel time of the PF. Postural stability is the most representative test among a variety of static balance tests. A higher postural stability level represents a stronger capability of the experimental subject to avoid falling down. In the static PF scenario, the experimental subject starts by standing on both legs and as the PF approaches the avatar in the game scene the subject needs to raise one leg up to fit the shape of the PF. The reaction time, which is the amount of time it takes the player to make a move as the PF approaches, and the size of the PF are used as parameters to study the subjects balance control performance. The experimental subject is expected to be able to maintain standing on one leg for 5 seconds to simulate this common posture in our daily life, for instance, while putting on pants. For the dynamic PF, the leg-raising angle and leg-raising rate are the main parameters to be monitored. Similar to the static PF scenario, the player starts by standing on both legs and the approaching PF prompts the experimental subject to raise their leg to the appropriate height of the PF. The player must complete the movement from a two-leg to one-leg stance within a given time limit. Generally speaking, In the static posture frame you have either one or two seconds to go from a two legged stance to a raised leg stance with a small or large frame, and maintain the one legged posture for five seconds; In the dynamic posture frame, you have one or two seconds to go from a two legged stance to either 45 degree or 90 degree leg lift. Players can practice their agility and balance, and their balance ability and postural reaction is evaluated. The design of the game parameters is described in detail below:

1. Offset of the posture frame: This denotes the distance between the posture frame boundary and the avatar, which might have a negative effect on the stability of the player's balance control due to a sense of tension induced by the posture frame, as shown in Figure 3. In this study, the offset of the posture frame is designed with two levels, a wider frame of 10 units (a), and a narrower frame of 5 units (b).
2. Travel time of the posture frame: This parameter denotes the time that the PF approaches the avatar since its appearance, which is designed to examine the impact of the player's reaction to the appearance of the posture frame on the movements, as shown in Figure 4. Two values are used in this study, 1 and 2 seconds, respectively.
3. Leg-raising angle: The leg-raising angle is determined by the height of an obstacle when the individual steps over it, as depicted in Figure 5. This study has two values for the leg raising angle, 45 and 90 degrees, respectively.
4. Leg-raising rate: This parameter denotes the time interval from standing on two legs to one leg. When the raising time is short, the player needs to reach the required one-leg posture quickly, as the leg-raising rate is relatively high, whereas when the raising time is longer, the player has to raise the leg more slowly and maintain their balance prior to passing through the PF.. Because under normal circumstances the average person needs to take approximately 1.7 seconds to raise their leg, the parameters in this work are designed to be 1 and 2 seconds, respectively.

### Software architecture

**Figure 6:**
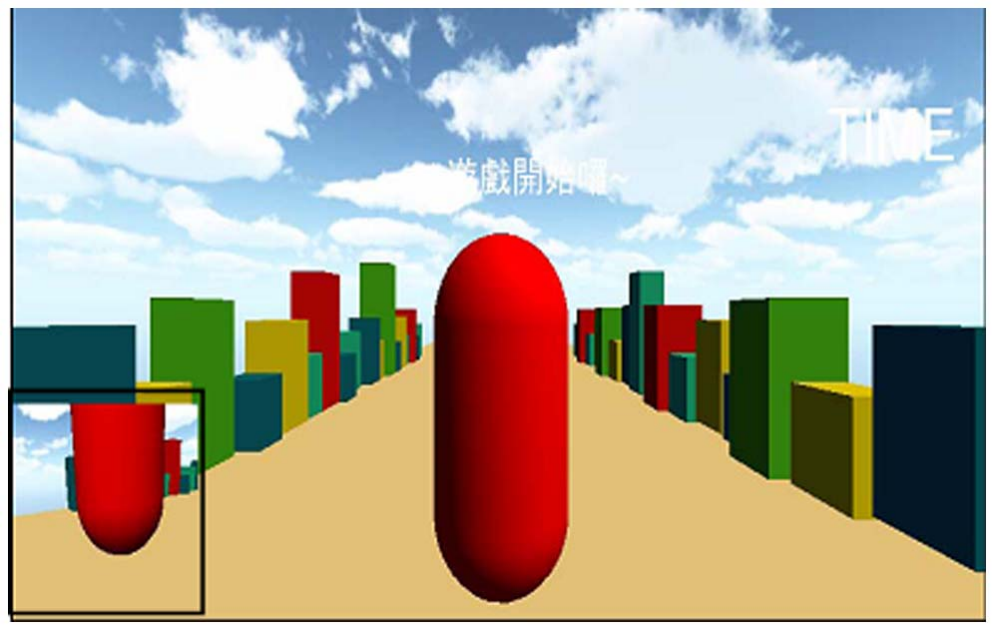
Virtual scene of the exergame for one-leg stand balance training and partial side view.

This work uses the 3D modeling software, Cinema 4D developed by MAXON Computer Inc., and the 3D interactive software, Unity 3D [37], for modeling and integration, which is divided into three parts: modeling of virtual game scenes, modeling of the avatar in the virtual environment, and integration of the avatar with Kinect. The modeling of the game scenes mainly includes adding virtual objects, designing the sky, and integrating some simple interactive programs, such as random appearance of virtual objects in the environment, time of play, and detection of collisions. In addition, sound effects are added, such as some blast sounds for collisions, to create a real environment as if the player is personally in the scene. A partial side view of the avatar is also provided and shown at the bottom left corner of the screen for the player to observe the movements of the avatar from a different angle, as depicted in Figure 6.

**Figure 7:**
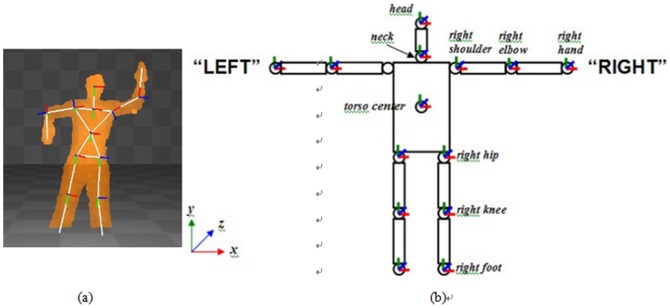
(a) Diagram of skeletal data collected by OpenNI. (b) Name and position of each articulation point. (Reference source: http://kheresy.wordpress.com/2011/01/28/detecte_skeleton_via_openni_part1/).

**Figure 8:**
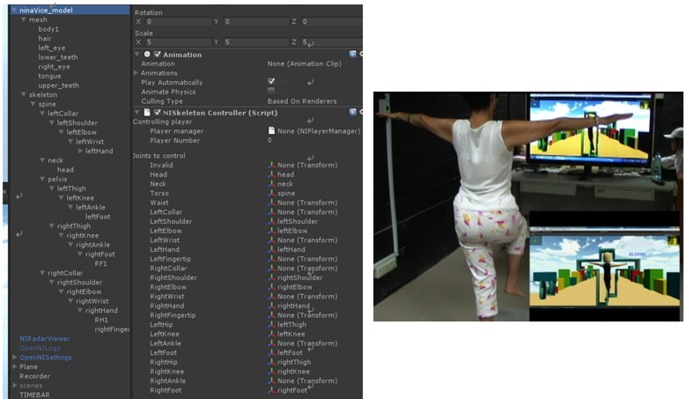
Scripts for matching skeletal information of the avatar and diagram of avatar control via Kinect by the player.

Moreover, in order to enable Kinect to control the avatar, the driver software, KinectSDK developed by OpenNI [38] is first installed, which is used to collect the data from fifteen articulation points of the experimental subject, as shown in Figure 7(a) and 7(b). After Kinect has received the data from fifteen articulation points, OpenNI will save these data points temporarily in the database. The interactive program developed in Unity3D will later call upon these data. Finally, Unity3D will match the collected data points with the skeletal system that has been already developed for the avatar, as shown in Figure 8, enabling the experimental subject to control the avatar in the virtual scene.

This study uses AMTINetForce to measure and collect the center of pressure (COP) data to evaluate the intensity level of exercise in terms of the player's balance control skill. The sample rate for monitoring the COP data is 100 Hz in this study.

### Hardware architecture

The hardware equipment used in this study includes one Microsoft Xbox360 Kinect with the motion caption system embedded. Kinect is able to collect original color images, 3D depth images, and audio signals. It can detect the player's actions based on 3D depth sensing technology, and control the avatar to make movements in the game scene and to interact with the posture frame. Other equipment includes a 55″LCD TV, a computer to install all the Kinect-related software including Unity3D, OpenNI, Prime Sense, and NITE, a computer to install AMTINetForce for driving the force plate, and a 6-axis AMTI force plate to study objective and gameplay-based player experience.

### Experimental design

This study aims to investigate the impact of different designs of game parameters on player experience from the perspective of effectiveness of the dual flow model based on an exergame of balance training. The exergame is played via Kinect, in which the player needs to maintain their balance while standing on one leg. Player experience is evaluated from both an objective perspective, based on data collected by the force plate, and a gameplay-based perspective. The experimental design is based on analysis from the viewpoint of a within-subjects design and comparative analysis between different parameter settings of static and dynamic PFs.

### Ethics committee

This study was approved in the Research Ethics Committee of National Taiwan University and has been classified as expedited on August 28 2012. The committee is organized under, and operates in accordance with, Social and Behavioral Research Ethical Principles and Regulations of National Taiwan University and governmental laws and regulations. All subjects provided written informed consent to participate in the study prior to entering the laboratory. We did not conduct research outside of our country of residence.

### Subjects

Twenty-three healthy individuals, 12 male and 11 female, aged between 21 and 30 participated in this study. All of the participants needed to get a full score in the Berg Balance Scale test to reduce the risk of falling down, and they could have no medical history regarding the central nervous system or relevant skeletal and muscular diseases to obtain a standard for evaluating the intensity level of exercise. All experimental subjects signed consent forms prior to the experiments.

### Procedure

**Figure 9:**
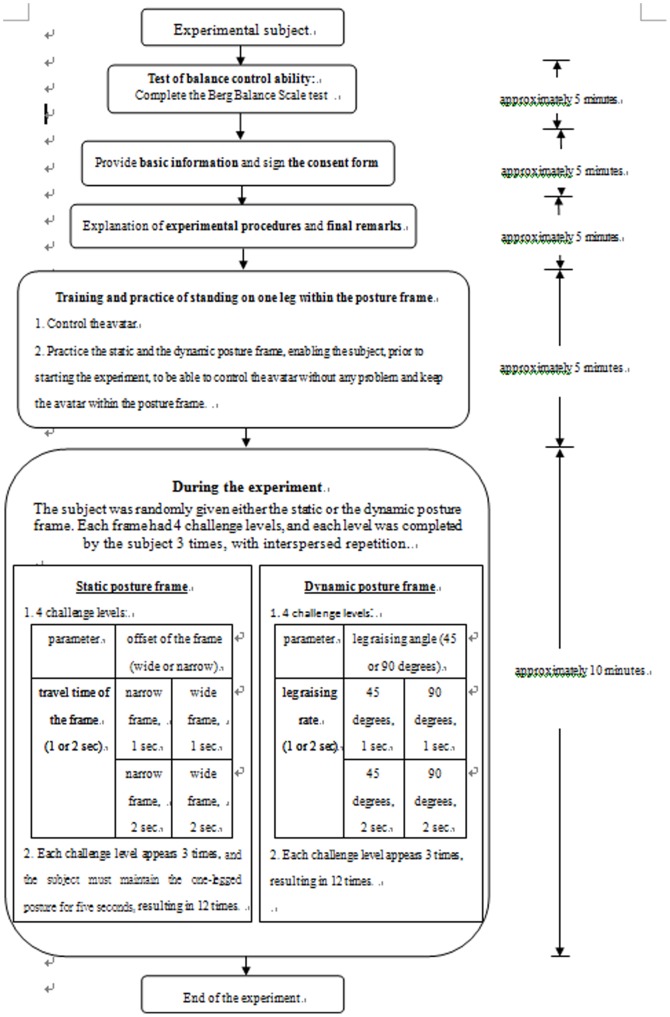
Flow chart of the experiment.

The flow chart of the experimental procedure is illustrated in Figure 9. The experimental subjects first had to evaluate their balance control abilities based on the Berg Balance Scale. After ensuring that they had no defects in balance control, subjects needed to provide some basic information and sign the consent form, followed by an explanation of the experimental procedures and final remarks. Prior to starting the experiment, the subjects were trained on how to control the avatar in the exergame, and were given an opportunity to become familiar with the required movements.

The experimental environment was divided into static and dynamic posture frames, respectively. Both of the frames had two game parameters, each of which had two different levels, resulting in four different challenge levels for each PF scenario. Each experimental subject attempted each level three times repetitively. The researcher first chose the PF from either a static or a dynamic frame, in a random manner. The selected PF then appeared 12 times, each of which was associated with a certain challenge level randomly selected from the 4 aforementioned challenge levels, resulting in each challenge level being played 3 times. Each experimental subject had to play the game 24 times (3 times for each of the 4 challenge levels for 2 PFs) in order to finish the experiment. All the design values for both PFs were interspersed and randomly assigned within the aforementioned 24 challenges, in order to avoid the learning effect.

### Player experience

This work focuses on studying both objective and gameplay-based player experience of healthy young adults, and evaluates the intensity level of exercise on the body using different game parameters, which might have an impact on the balance control ability of the player, in order to design training standards.

### Objective - Center of Pressure

**Figure 10:**
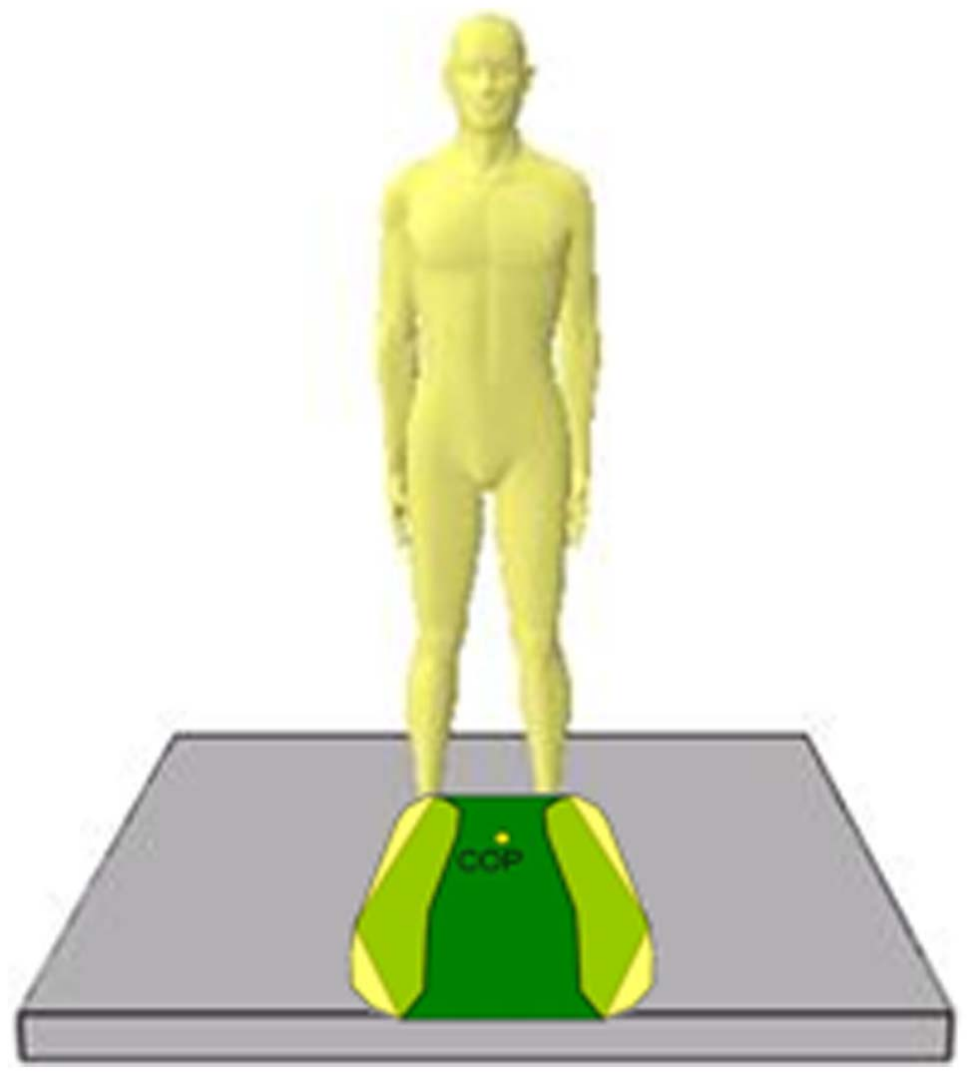
Schematic diagram of COP (Zhuo, 2007).

Balance control ability refers to the ability to stabilize and maintain the center of mass (COM) on the base of support (BOS) [39]. The two most commonly used measurements to evaluate static balance ability are measurements of the center of gravity (COG) and the center of pressure (COP), respectively. In this study, COP is used as the measurement to analyze the balance ability of the subject. COP refers to the center of pressure transmitted down to the ground due to COG while the subject is standing on two legs, as illustrated in Figure 10. Odenrick [40] compared COG with COP to study which indicator can better reflect the balance control ability. Results have shown that variations in COG are smaller than changes in COP. Odenrick stated that COP displacement is more significant because the experimental subject tries to maintain his or her COG within the sustainable threshold of balance. Our work takes advantage of COP as being an indicator of the balance control ability, and simultaneously uses the force plate, which serves as an objective and accurate tool in measuring postural stability, to achieve quantification of postural control.

Previous studies mostly used the motion capture system [10]–[12] and force plate [13], [22], [24], [25] to collect data regarding the displacement of the center of pressure and body-sway intensity. The motion capture system monitored the body movements of the subject in the X-Y-Z space of the virtual environment, such as displacement, angle changes, and spin of head, main body, and feet articulation points. On the other hand, the force plate focused on collecting horizontal displacement in the 2-dimensional X-Y space, such as displacement along the anterior-posterior and mediolateral directions. Although the motion capture system is able to collect 3-dimensional displacement of the subject and calculate the sway intensity of the body, this study focused on collecting 2-dimensional horizontal displacement of the body while the experimental subject stood still on one leg within the posture frame and assessed the body sway intensity for different frame parameters. In addition, when studying the balance performance by evaluating postural stability, the impact of different sensorimotor inputs on balance performance can be seen by measuring displacement of the plantar supporting surface in response to virtual scene changes while the subject is on the force plate [41]. In order to study the objective player experience, this study used the force plate to monitor displacement of the center of pressure to quantify body-sway intensity and posture stability.

To evaluate balance control ability while standing on one leg, many existing works have used measurements such as total sway area, mean displacement in the anterior-posterior direction, mean displacement in the mediolateral direction, and total excursions [33], [42]–[44]. Verhagen et al. [32] claimed that mean displacement distance along the anterior-posterior/mediolateral directions is represented by the mean COP displacement along the corresponding direction per unit time, which can be used as a performance metric for assessment of the ability of balance control. The smaller the displacement measured the higher stability of balance ability it represents. Paillard and his colleagues [45] stated that total excursion, defined as total COP displacement per unit time, is also related to balance control ability and can be used as a measurement to evaluate balance stability and muscle strength. A smaller total excursion indicates a better performance of balance control. Total sway area denotes the COP sway area per unit time, and is related to the body's proprioception and visual function. Similar to the aforementioned total excursion, a smaller sway area indicates a better performance of balance control.

**Figure 11:**
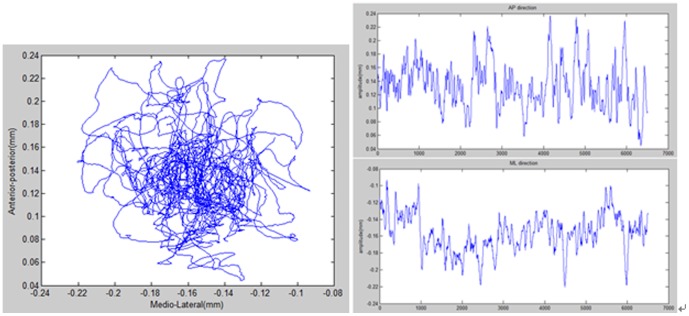
Diagram of COP sway trajectory (left), resolved AP component of the sway trajectory (upper right) and resolved ML component of the sway trajectory (lower right).

Accordingly in this study, four performance metrics were evaluated, namely, mean distance-anterior posterior (MDIST-AP), mean distance-medial lateral(MDIST-ML), sway area(AREA-SW), and total excursions (TOTEX), to assess the subject's balance control performance in the case of various designs of game parameters. Forces and moments along the x, y, and z direction, namely, Fx, Fy, Fz, Mx, My, and Mz, were collected by the force plate. These data were preprocessed, and manipulated in Matlab to calculate trajectory directions of body sway based on equations (1) and (2), which resolved the COP trajectory into Py (equivalent to AP) and Px (equivalent to ML) rectangular components. Finally, the data was analyzed to assess the balance control ability of the experimental subject, as illustrated in Figure 11. In the equation below, P represents the coordinate of the x, y, and z axis, F represents the force; M represents the moment.
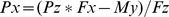
(1)

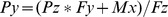
(2)


Based on the study by Perito et al. [46], a sample rate of 100 Hz with a 30-second sample time interval will result in 3000 data points. Because there was some discrepancy in the position where each experimental subject stood on the force plate, each collected data point needed to be calibrated prior to being manipulated and analyzed in terms of the four aforementioned COP metrics. As formulated in equations (3) and (4), the mean displacements along the anterior-posterior and mediolateral directions were calculated, in which AP_0_[n] and ML_0_[n] represented the raw COP data along the AP and ML directions at time point n, respectively.
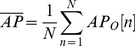
(3)

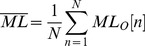
(4)


Zero-point correction was then performed by subtracting the raw COP data with the mean value that was obtained previously, as formulated in equations (5) and (6), where N denotes the total number of data points.
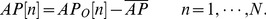
(5)

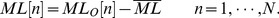
(6)


#### Mean distance (MDIST)

The AP and ML displacement with respect to the zero point, represented by RD, was first calculated by Equation (7). The mean of RD, denoted as MDIST, was then obtained from equation (8), where N is the total number of data points.
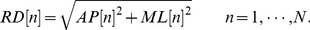
(7)

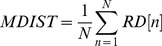
(8)


#### Mean distance-anterior posterior (MDIST_AP_)

MDIST_AP_ denotes the mean displacement along the anterior-posterior direction, which was calculated using equation (9).
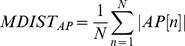
(9)


#### Mean distance-medial lateral (MDIST_ML_)

MDIST_ML_, similar to MDIST_AP_, represents the mean displacement in the mediolateral direction, which was obtained from equation (10).
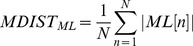
(10)


#### Sway area (AREA_SW)

The total COP sway area was calculated using equation (11), where AP [n+1] and ML[n+1] represent the AP and ML data at the next time point, and T is the length of the time frame.
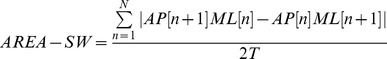
(11)


#### Total excursions (TOTEX)

The total excursion, represented by TOTEX, was calculated using equation (12).
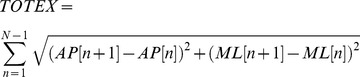
(12)


### Gameplay-based - number of collisions

**Figure 12:**
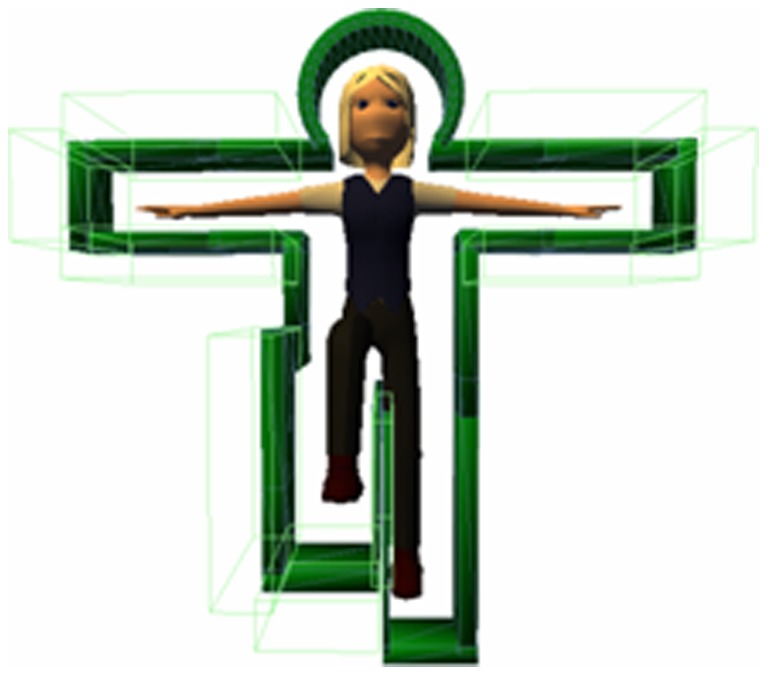
Detection of collisions between the avatar and the posture frame.

Because the most obvious postural sway occurs at both arms and the right leg while the subject is standing on their left leg, this study deployed collision sensors for both arms and the right leg, with a sample rate of 0.1 second, to monitor if there was any collision between the avatar and the posture frame. The number of collisions was then collected to quantify the body sway level of the experimental subject, acting as the indicator for evaluation of the gameplay-based player experience, as illustrated in Figure 12. A score display provides feedback to the user concerning his or her current performance.

### Statistical Analysis

Statistical analysis was carried out for the tests of both objective and gameplay-based player experience. We used Statistical software SPSS V12.

### Objective player experience

As previously mentioned, this study aims to examine the impact of different designs of posture frame parameters on the balance control ability of the experimental subject, based on collecting COP information to evaluate the actual body-sway level of the participant. Force and moment data collected by the force plate, namely, Fx, Fy, Fz, Mx, My, and Mz, were preprocessed and manipulated in Matlab to calculate all the COP performance metrics, including MDIST-AP, MDIST-ML, AREA_SW, and TOTEX.

When all the aforementioned COP metrics were calculated, ANOVA based on repeated measures was applied. Software SPSS was utilised in this work to statistically study repeated measures under the general linear model (GLM), and to analyze the impacts of the design parameters for both static and dynamic posture frames on the COP data of the experimental subjects. This aimed to help obtain a good knowledge of the intensity level of the exercise that the player is able to handle, enabling a better design of the difficulty level of exergames, especially for those with specific training purposes.

### Gameplay- based player experience

As previously discussed, both the static and dynamic posture frames have four different designs of challenge levels. With the static posture frame, collision sensor nodes are located at both of the arms and the right leg, whereas for the dynamic frame, only the right leg collisions are taken into account because this part of the study is focused on assessing balance control performance of the subject while raising the leg.

To evaluate the gameplay-based player experience in terms of the number of collisions and investigate the impact of game parameters on player intensity, the experimental subject needed to repeat each test case with a specific design of posture frame parameters three times. The study used statistical analysis including the mean value as well as the standard deviation, based on the number of collisions measured from three attempts. Repeated measures of ANOVA were carried out to analyze the impact of frame parameters on the subject for both static and dynamic posture frames in order to establish how the game parameters affect the intensity level of the exercise, enabling a better design of step-by-step difficulty levels of exergames and improving the effectiveness of exergames with training purposes.

## Results

### Objective player experience

Table 1 and 2 show the COP results with respect to different design parameters of the static and dynamic posture frames, respectively. Each COP metric, namely MDIST-AP, MDIST-ML, AREA_SW, and TOTEX, is represented by the mean and the standard deviation obtained from statistical analysis based on the results from 3 repetitive runs for each test case.

**Table 1:** COP results for different design parameters of the static posture frame.

	Smaller frame width with a frame travel time of 1 sec	Larger frame width with a frame travel time of 1 sec	Smaller frame width with a frame travel time of 2 sec	Larger frame width with a frame travel time of 2 sec
MDIST-AP	0.77±0.25	0.70±0.18	0.97±0.25	0.94±0.29
MDIST-ML	1.98±1.16	1.99±1.16	1.94±1.47	1.72±1.32
TOTEX	53.98±15.57	53.68±17.28	53.68±16.32	51.52±17.87
AREA_SW	0.07±0.06	0.06±0.05	0.06±0.05	0.06±0.02

Unit: mm (mean ± standard deviation).

**Table 2:** COP results for different design parameters of the dynamic posture frame.

	a leg raising rate of 1 sec with a leg raising angle of 45 degrees	a leg raising rate of 1 sec with a leg raising angle of 90 degrees	a leg raising rate of 2 sec with a leg raising angle of 45 degrees	a leg raising rate of 2 sec with a leg raising angle of 90 degrees
MDIST-AP	0.47±0.25	0.46±0.23	0.52±0.23	0.51±0.21
MDIST-ML	0.89±0.69	0.95±0.75	1.18±0.84	1.11±0.83
TOTEX	13.93±3.79	14.46±5.29	24.94±5.46	25.22±6.09
AREA_SW	0.03±0.02	0.01±0.01	0.04±0.02	0.04±0.03

Unit: mm (mean ± standard deviation).

Tables 3 and 4 provide the analysis results showing the impact of different designs of posture frame parameters on COP displacement of the experimental subject, which were obtained based on repeated measures of ANOVA. Based on the ANOVA analysis, no interaction among the factors for all of the COP metrics was observed. As a result, each factor was analyzed in an independent manner to study the impact of the game parameters. The results indicate that, for the static posture frame, only MDIST-AP was affected by the frame travel time (p = 0.000<0.05), while the other metrics were not related to the offset of the frame or the frame travel time. For the dynamic posture frame, an impact of the leg-raising rate on MDIST-ML (p = 0.003<0.05) and TOTEX (p = 0.000<0.05) was observed.

**Table 3:** P-value of COP metrics for the static posture frame.

	Offset(offset of the frame)	Time(travel time of the frame)	Off×Speed
MDIST-AP	0.179	0.000*	0.416
MDIST-ML	0.386	0.347	0.328
TOTEX	0.435	0.480	0.445
AREA_SW	0.276	0.060	0.212

* p-value<0.05.

**Table 4:** P-value of COP metrics for the dynamic posture frame.

	Angle(leg-raising angle)	Speed(leg-raising rate)	Angle×Speed
MDIST-AP	0.687	0.152	0.957
MDIST-ML	0.979	0.003*	0.312
TOTEX	0.308	0.000*	0.749
AREA_SW	0.479	0.068	0.283

* p-value<0.05.

### Gameplay-based player experience

Every experimental subject needed to repeat each test case three times, the results of which were used to calculate the mean as well as the standard deviation, as shown in Tables 5 and 6. Repeated measures of ANOVA were then used to analyze the impact of the different designs of frame parameters on the subject deviation, for both the static and dynamic posture frames. Based on the ANOVA analysis, no interaction was found among factors for all of the collision detection nodes. Therefore, it is reasonable to treat each factor independently when studying the impact of game parameters. Analyses of the results are provided in Tables 7 and 8. The results show that for the static posture frame, the frame travel time had an impact on all three detection locations, the left arm shows a p-value of 0.000(p<0.05), the right arm shows a p-value of 0.000(p<0.05), and the right leg has a p-value of 0.000(p<0.05); the offset of the PF shows that the left arm and right leg are significant, with the left arm p-value of 0.013(p<0.05), and the right leg p-value of 0.027(p<0.05). For the dynamic posture frame, both the leg-raising rate (p-value = 0.029<0.05) and leg-raising angle (p-value = 0.001<0.05) had an impact on the subject, while no impact of the offset of the frame was observed.

**Table 5:** Number of collisions for different design parameters of the static posture frame.

	Smaller frame width with a frame travel time of 1 sec	Larger frame width with a frame travel time of 1 sec	Smaller frame width with a frame travel time of 2 sec	Larger frame width with a frame travel time of 2 sec
left arm	5.54±4.59	3.68±2.38	0.78±1.14	0.36±0.78
right arm	5.72±5.67	4.16±2.85	1.16±2.37	0.58±1.08
right leg	5.80±3.86	3.86±4.59	1.68±2.41	0.78±1.58

Unit: mm (mean ± standard deviation).

**Table 6:** Number of collisions for different design parameters of the dynamic posture frame.

	a leg raising rate of 1 sec with a leg raising angle of 45 degrees	a leg-raising rate of 1 sec with a leg-raising angle of 90 degrees	a leg-raising rate of 2 sec with a leg-raising angle of 45 degrees	a leg-raising rate of 2 sec with a leg-raising angle of 90 degrees
right leg	3.78±1.78	6.28±2.34	6.94±4.48	9.62±5.29

Unit: mm (mean ± standard deviation).

**Table 7:** P-value of the number of collisions for the static posture frame.

	Offset(offset of the frame)	Speed(travel time of the frame)	Off×Speed
left arm	0.013*	0.000*	0.134
right arm	0.062	0.000*	0.424
right leg	0.027*	0.000*	0.334

* p-value<0.05.

**Table 8:** P-value of the number of collisions for the dynamic posture frame.

	Angle(leg-raising angle)	Speed(leg-raising rate)	Angle×Speed
right leg	0.001*	0.029*	0.708

* p-value<0.05.

## Discussion

This study combined a Kinect based exergame with balance training by maintaining a one-leg stance posture. Results in terms of detection of the number of collisions of both arms and the right leg with the frame show that the number of collisions varied significantly according to the frame parameter that was measured, namely, the travel time of the frame or the leg-raising rate and angle. In terms of COP metrics, a significant impact of the travel time of the frame on MDIST-AP was observed for the static posture frame, and for the dynamic frame, the impact of the leg-raising rate on MDIST-ML and TOTEX was found to be significant. Because the experimental subject needed to maintain the one-legged posture for five seconds in the case of the static posture frame, the number of collisions was always large regardless of the design of frame parameters. Compared to the dynamic posture frame, where the experimental subject needed to raise the leg to match the movement of the frame, the number of collisions appeared to be greater in the dynamic condition. As a result, a significant impact of frame parameters was observed based on statistical analysis. Overall, both the travel time of the frame and the leg-raising rate were found to have an impact on both the number of collisions and the trajectory of COP displacement, while the leg-raising angle was only observed to have an impact on the number of collisions. Overall, the COP metrics were greater in the dynamic condition, which demonstrates that the subjects found the dynamic postures easier than the static postures.

In terms of the number of collisions for the static posture frame, regardless of where the collision occurred (either of the arms or the right leg), the best stability of balance control was observed when a larger frame was offset with a frame travel time of 2 seconds. This was followed by a smaller frame offset with a travel time of 2 seconds, a larger frame offset with a travel time of 1 sec, and a smaller frame offset with a travel time of 1 second. As all the experimental subjects were healthy young adults with a full score on the Berg Balance Scale test, the obtained results can be used as standard not only for balance training but also to design the exergame with different difficulty levels for various training modes.

In terms of the number of collisions for the dynamic posture frame, the best performance in balance control was observed with a leg-raising rate of 1 second and a raising angle of 45 degrees, while the worst performance was obtained with a leg-raising rate of 2 seconds and an angle of 90 degrees. This indicates that the difficulty level is relatively lower when the balance needs to be maintained for a shorter time frame. However, in the static posture frame, the opposite is true. Because of the design mechanism in the static condition, the subject needs to ‘aim’ for the same shape as the posture frame to pass through it; therefore, the subjects demonstrated a better performance in the case of a longer reaction time. However, in the dynamic condition, the subjects are required to follow the movement of the frame without incurring any collisions. Consequently, in order to increase the intensity level of the exercise, the reaction time designed for the static posture frame in the exergame might be extended.

This study is based on variations of multiple parameter combinations because a simple parameter design tends to make the player feel bored and lack adherence. When the difficulty level can be easily determined with a single parameter, multiple parameter combinations make it much less straightforward. For instance, in terms of the number of right-foot collisions with a dynamic PF, the best balance performance was obtained with a leg-raising rate of 1 second and an angle of 45 degrees, and the worst case was with a 2-second leg-raising rate and a 90-degree raising angle. Based on the aforementioned findings, the difficulty level is lower when maintaining the balance for a shorter period. From the viewpoint of design with a single parameter, either the leg-raising rate or raising angle has an impact on the difficulty level of maintaining balance. From the exergame design point of view, however, the player might easily respond to variations of a single parameter in a mission. Therefore, to improve the effectiveness and adherence of exergames and to make the player feel challenged, a combination of various game parameters, instead of single parameter, might be considered in the exergame design. This work is based on a multi-parameter design. This finding shows a better performance is achieved when a shorter reaction time is provided. This is not intuitively predictable, but is realized through our approach based on analysis of variations of parameter combinations.

It is worth noting that an inconsistency was obtained between the results for the objective and gameplay-based player experience. For the static posture frame, demonstrated in terms of the index MDIST-AP, on which the game parameters appear to have a significant impact, a travel time of 1 second resulted in better balance performance, while a better performance in terms of the number of collisions was found with a 2-second PF travel time. In the static scenario, the PF approaches the avatar, or the user, and the results show that a shorter PF travel time leads to a better balance stability. However, it was also found that a longer travel time resulted in a better performance in terms of how the player moves his or her body to go through the PF without any collisions. The score display provides feedback to the user concerning his or her current performance. By comparing objective and gameplay-based player experience using COP index, it possible to study the balance performance of the experimental subject, or equivalently, the difficulty level from the viewpoint of the player. Gameplay-based performance helps to motivate the player to avoid collisions with the frame to obtain a higher score, which enhances the self-satisfaction of the player and the adherence of the exergame. These findings might be caused by insufficient consideration being given to the process of exergame design, as the exergame has to take both enjoyment and the purpose of training into account.

Otherwise, for the static posture frame, no significant impact was observed in terms of all COP metrics in the offset, while the impact is significant in terms of the number of collisions. For the dynamic posture frame, the leg-raising angle does not have a significant impact on COP displacement, but a strong impact on the number of collisions was obtained. The aforementioned findings can be explained by the fact that in both conditions, the experimental subject passively waits for the posture frame to approach the body, resulting in no impact on COP displacements, but dynamically raising the leg, produces significant results in terms of the number of collisions. In the static condition, more sway correlates with fewer collisions, whereas in the dynamic condition, more sway correlates with more collisions. In both conditions, a longer approach time correlated with more sway. In addition, from the examples included there appears to be some difference in the pose of the dynamic posture versus the static posture. In the dynamic postures it appears the knee bend occurs more laterally, whereas in the static condition the knee bend appears to be in front of the subject. This result shows that maintaining balance with lateral bends is harder than with forward bends.

For the static PF, the offset of the PF has little impact on balance control performance in terms of COP metrics, whereas from the perspective of gameplay-based player experience, the number of collisions obtained from the feedback of the exergame has a significant impact on the performance because the experimental subject cares about his or her score. These results are consistent with the findings in the work by Keshner et al. [12], in which the impact of visual changes on postural reactions was investigated. When the subject is standing still and the virtual scene is moving or changing, the virtual scene puts an impact on the head and the main body of the subject, but relatively little impact is found on the foot of the subject in terms of the amplitude and frequency. This could be explained by the fact that the subject tries to control the stability of their feet in order to maintain their balance on the floor. Meanwhile, this study finds a significant impact due to game parameter variations on the number of collisions but little impact on the COP metrics. From the viewpoint of dual flow model, Sinclair et al. [4] stated that monitoring the events, actions, and responses of an exergame player during game play is one potential area for investigation. In addition, from the perspectives of both objective and gameplay-based player experience, it is found that subjects are more concerned about their scores and appear to be much less concerned about their performance represented by the COP metrics. These findings indicate that different evaluation approaches for player experience from the viewpoint of exergame design could result in different outcomes. In addition, it is worth noting that, although there is no significant difference among the result with respect to each of the four COP metrics measured in this study, different results might be obtained in the case of different metrics selected by other researchers.

Previous works have used virtual reality technology combined with a motion capture system to evaluate the balance control ability of the subject while standing on one leg within a posture frame [34]–[36]. This study extends the existing studies by replacing the motion capture system with Kinect, which saves the subject a lot of time and effort in putting on the clothes specially built to support motion caption and making a calibration of motions with the software. This study applies Kinect to enable intuitive interactions between the player and virtual scenes, which adds fun and objectiveness to exergames with training purposes. In particular, the subject's balance performance representing his or her body intensity in the case of different parameter designs (i.e., difficulty levels) is being monitored, and simultaneously, the player is able to enjoy receiving immediate feedback on his or her performance in the exergame. COP metrics are measured to evaluate the balance capability of the subject, which is a convincing approach in terms of the effectiveness and a well-founded way for studying body intensity of the subject. Some of the previous studies have used a force plate to record COP information, and transformed COP to corresponding COG data [47]–[48]. In this study, we used a force plate to obtain the COP data, but we did not transform COP to COG information as in the work by Rougier [48], in which various mathematical relationships between COP and COG were proposed, and the relationship among COP, COG, and the subject performance was discussed. Our future work might include transformation between COP and COG data and address their relationship with the performance of the experimental subject. These findings regarding the impact of different designs of game parameters on the subject's performance would be used as helpful feedback in supporting future exergame design and customization. The expected game platform could be used in both health care centers and individual homes because of its low cost and ease of use.
